# A Novel Synthesis of the Oxazolidinone Antithrombotic Agent Rivaroxaban

**DOI:** 10.3390/molecules190914999

**Published:** 2014-09-18

**Authors:** Jianyong Yuan, Kai Liu, Lun Li, Yong Yuan, Xuelei Liu, Yanwu Li

**Affiliations:** Pharmacy College, Chongqing Medical University, Chongqing 400016, China; E-Mails: enediyne@163.com (J.Y.); lkai2yyuan@163.com (K.L.); manund28@126.com (L.L.); drugsyy2013@163.com (Y.Y.); xueleiliua@126.com (X.L.)

**Keywords:** rivaroxaban, oxazolidinone, Goldberg coupling reaction

## Abstract

A facile synthetic route of rivaroxaban has been developed. Using commercially available (*R*)-epichlorohydrin and bromobenzene as the starting materials, rivaroxaban was obtained in 39% overall yield using a Goldberg coupling as the key step. The synthetic route represents a convenient procedure for the production of rivaroxaban.

## 1. Introduction

**Figure 1:**
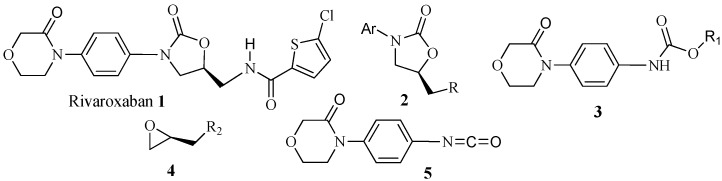
Structures of rivaroxaban (**1**) and compounds **2**–**5**.

Factor Xa is a coagulation factor that plays a central role in the coagulation process [1,2,3]. Rivaroxaban (**1**, Figure 1), a member of a new class of potent Fxa inhibitors, is a novel oxazolidinone derivative that provides a simple, fixed-dose regimen for the prevention and treatment of thromboembolic disorders [4]. The introduction of rivaroxaban into the market has led to the need for its efficient production. For these reasons, much attention has been given to the synthesis of rivaroxaban and several synthetic methods for this product were developed. Most reported methods for the synthesis of **1** involve the construction of the 5-aminomethyl-3-aryl oxazolidinone **2** as a key step [5,6,7,8,9,10]. A common strategy for the preparation of **2** begins with the deprotonation of the aryl carbamate **3** with *n*-BuLi and treatment of the resulting anion with (*R*)-glycidylbutyrate (**4**) to give the corresponding 5-(*R*)-hydroxymethyloxazolidinone. Opening of *N*-glycidylphthalimide with 4-(4-aminophenyl)morpholin-3-one and then ring closure with *N*,*N'*-carbonyldiimidazole also provided the oxazolidinone, which was subjected to deprotection and acylation to obtain rivaroxaban. In an alternative strategy, the aryl oxazolidinone could be obtained from aryl isocyanides **5** and (*R*)-glycidylbutyrate (**4**), but the preparation of aryl isocyanates from aryl amines is cumbersome. Other methods reported in literature involve metal-catalyzed coupling reactions between aryl halides and oxazolidinones [11,12,13,14]. Recently, several different research groups reported the application of the Goldberg coupling reaction [15] for the amidation of aryl halides. In an attempt to overcome the limitations of the traditional syntheses of rivaroxaban, a convenient synthetic method involving the Goldberg coupling reaction of commercially available (*R*)-epichlorohydrin and aryl bromide as the key step is reported in this paper.

## 2. Results and Discussion

**Scheme 1:**
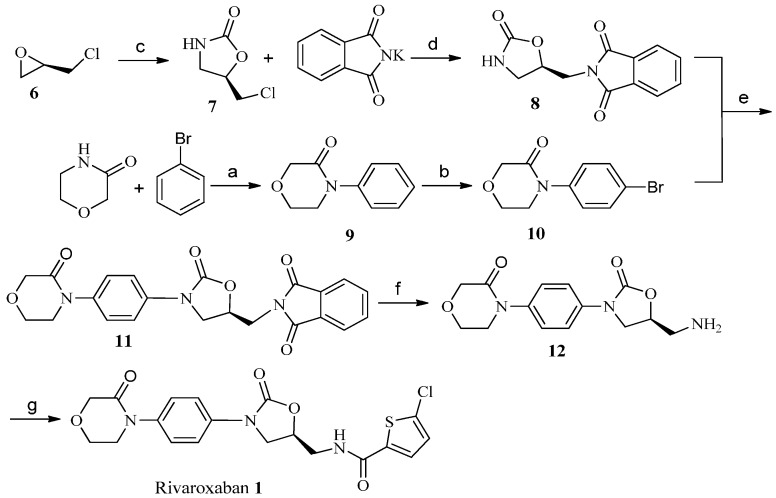
Synthesis of rivaroxaban (**1**).

After a detailed survey of the previous preparations of rivaroxaban (**1**), we chose as the starting material for our synthesis the commercially available chiral (*R)*-epichlorohydrin (**6**) which was reacted with NaOCN to afford (*R*)-chloromethyl-2-oxazolidinone (**7**) as a key chiral intermediate (Scheme 1). 

*Reagents and conditions*: (**a**) CuI (5 mmol %), (*Z*)-1,2-diaminocyclohexane (10 mol %), dioxane, K_2_CO_3_, 110 °C; (**b**) NBS, DMF, r.t.; (**c**) NaOCN, 60 °C; (**d**) potassium phthalimide, DMF, 80 °C; (**e**) CuI (5 mmol %), (*Z*)-1,2-diaminocyclohexane (10 mol %), dioxane, K_2_CO_3_, 110 °C; (**f**) hydrazine hydrate (80%), MeOH, reflux; (**g**) 5-chlorothiophene-2-carboxylic acid, thionyl chloride, Et_3_N, DCM, r.t.

## 3. Experimental Section

### General Information

Unless otherwise stated, all chemical reagents purchased (Aldrich, J&K Scientific Ltd., Beijing, China) were of the highest commercially available purity and were used without previous purification. ^1^H, ^13^C spectra were on a Bruker Avance 300 NMR spectrometer.

*(R)-5-(Chloromethyl)oxazolidin-2-one* (**7**). To a mixture of magnesium sulfate (12.0 g, 0.1 mol) and sodium cyanate (65.0 g, 1 mol) in water (500 mL) at 60 °C, (*R*)-epichlorohydrin (46.3 g, 0.5 mol) was added dropwise over 30 min, keep the mixture warm for 1 h. The water was removed under reduced pressure and then ethyl acetate (500 mL) was added. The mixture was filtered and the filter cake was washed with ethyl acetate (200 mL × 2), the combined ethyl acetate fraction were dried (Na_2_SO_4_) and concentrated to give a white solid, which was recrystallized from ethyl acetate to give 53.7 g (79%) of compound **7**, mp 64–65 °C. ^1^H-NMR (300 MHz, CDCl_3_): δ 3.48 (dd, 1H, *J* = 6.6, 4.6 Hz), 3.56 (t, 1H, *J* = 6.6 Hz), 3.80 (dd, 1H, *J* = 8.9, 3.7 Hz), 3.89 (dd, 1H, *J* = 8.9, 3 Hz), 4.84 (m, 1H), 7.62 (s, 1H), 7.88 (m, 2H). ^13^C-NMR (75 MHz, CDCl_3_): δ 40.8, 43.8, 73.4, 168.1.

*(R)-2-((2-Oxazolidin-5-yl) methyl) isoindoline-1,3-dione* (**8**). A solution of **7** (27.1 g, 0.20 mol) and potassium phthalimide (40.7 g, 0.22 mol) in DMF (500 mL) was heated to 80 °C for 12 h. The reaction was cooled to ambient temperature, diluted with water (2 L) and extracted with methylene dichloride (200 mL × 3), the combined organic layer was washed with saturated sodium chloride solution and then dried (Na_2_SO_4_). The solution was evaporated *in vacuo* to give a white solid, which was crystallized from ethyl acetate and petroleum ether to afford **8** (41.4 g, 84%) as a white solid, mp 195–197 °C. ^1^H-NMR (CDCl_3_, 300 MHz): δ 3.2 (dd, 1H, *J* = 6.6, 4.3 Hz), 3.72 (t, 1H, *J* = 6.6 Hz), 3.91 (dd, 1H, *J* = 10.6, 4.2 Hz), 4.10 (dd, 1H, *J* = 10.6, 5.2 Hz), 4.97 (m, 1H), 7.75 (m, 2H), 7.88 (m, 2H). ^13^C-NMR (75 MHz, CDCl_3_): δ 42.6, 46.3, 73.9, 158.2.

*4-Phenylmorpholin-3-one* (**9**). To a solution of morpholin-3-one (4.0 g, 39.6 mmol), bromobenzene (7.5 g, 47.8 mmol) and 1,2-diaminocyclohexane (0.9 g, 7.9 mmol) in 1,4-dioxane (150 mL), potassium carbonate (11.02 g, 79.2 mmol) and copper iodide (3.0 g, 15.84 mmol) were added. The mixture was stirred at 110 °C under N_2_ for about 36 h. The mixture was cooled to ambient temperature and filtered. The filtrate was concentrated *in vacuo*, diluted with water (500 mL) and extracted with ethyl acetate (50 mL × 3). The combined organic layers were dried (Na_2_SO_4_). The residue was purified by chromatography on silica gel column (ethyl acetate/petroleum ether = 5/1) to give compound **9** (5.8 g, 83% yield) as a white solid, mp 113–115 °C; ^1^H-NMR (400 MHz, CDCl_3_): δ 7.44 (t, *J* = 7.3 Hz, 2H), 7.46–7.29 (m, 3H), 4.36 (s, 2H), 4.05 (s, 2H), 3.78 (s, 2H). ^13^C-NMR (100 MHz, CDCl_3_): δ 166.7, 141.4, 129.4, 127.3, 125.6, 68.7, 64.2, 49.8.

*4-(4-Bromophenyl)morpholin-3-one* (**10**). A solution of NBS (2.4 g, 13.5 mmol) in DMF (15 mL) was added dropwise at room temperature to a solution of compound **9** (2.0 g, 11.3 mmol) in DMF (15 mL) over about 30 min. The resulting solution was stirred at room temperature for about 6 h. Water (200 mL) and ethyl acetate (50 mL) were added and the mixture was stirred at room temperature for about 30 min. The organic layer was separated, and the aqueous layer was extracted with ethyl acetate (50 mL × 3). The organic layers were combined and washed with brine (50 mL). The organic layer was dried with sodium sulfate (20 g) and concentrated. The residue was purified through silica gel column chromatography (ethyl acetate/petroleum ether = 1/2) to produce compound **10** (2.6 g, 90% yield) as a white solid. ^1^H-NMR (400 MHz, CDCl_3_): δ 7.53 (d, *J* = 8.3 Hz, 2H), 7.23 (d, *J* = 8.3 Hz, 2H), 4.33 (s, 2H), 4.02 (s, 2H), 3.74 (s, 2H). ^13^C-NMR (100 MHz, CDCl_3_): δ 166.7, 140.4, 132.5, 127.1, 120.7, 68.7, 64.2, 49.6.

*(S)-2-((2-Oxo-3-(4-(3-oxomorpholino)phenyl)oxazolidin-5-yl)methyl)isoindoline-1,3-dione* (**11**). The compounds **10** (2.0 g, 7.8 mmol), **8** (3.8 g, 8.6 mmol), potassium carbonate (2.2 g, 15.6 mmol), copper iodide (743 mg, 3.9 mmol) and 1,2-diaminocyclohexane (178 mg, 1.6 mmol) were mixed and solubilized in 1,4-dioxane (70 mL). The mixture was stirred at 110 °C in a clean flask under N_2_ for about 36 h. The mixture was cooled to ambient temperature, which was filtered and the filtrate concentrated *in vacuo*, diluted with water (50 mL) and extracted with CH_2_Cl_2_ (50 mL × 3). The combined organic layers were dried (Na_2_SO_4_). The residue was purified by chromatography on silica gel column chromatography (dichloromethane/methanol = 75/1) to produce compound **11** (2.6 g, 79% yield) as a white solid. ^1^H-NMR (400 MHz, CDCl_3_): δ 7.88 (m, 2H), 7.76 (m, 2H), 7.57 (d, *J* = 8.4 Hz, 2H), 7.34 (d, *J* = 8.3 Hz, 2H), 4.98 (m, 1H), 4.33 (s, 2H), 4.17–4.11 (m, 2H), 4.07–3.88 (m, 4H), 3.75 (s, 2H). ^13^C-NMR (100 MHz, CDCl_3_): δ 168.1, 166.9, 154.0, 137.5, 136.7, 134.6, 131.8, 126.3, 123.8, 119.3, 69.8, 68.7, 64.2, 49.8, 48.5, 40.9.

*(S)-4-(4-(5-(Aminomethyl)-2-oxazolidin-3-yl) phenyl) morpholin-3-one* (**12**). To a solution of compound (**11**) (800 mg, 1.9 mmol) in MeOH (50 mL), an 80% of hydrazine hydrate (1.19 g, 19 mmol) was added. The mixture was refluxed for 1.5 h. The mixture was filtered and the filter cake was washed with MeOH (5 mL × 3) and concentrated. The residue was purified by a short silica gel column, eluted with CH_2_Cl_2_/MeOH/NH_3_·H_2_O = 80/20/1 to give compound (**12**) (450 mg, 81% yield) as a white solid. ^1^H-NMR (400 MHz, DMSO): δ 7.59 (d, *J* = 8.3 Hz, 2H), 7.40 (d, *J* = 8.3 Hz, 2H), 4.62 (s, 1H), 4.19 (s, 2H), 4.08 (m, 1H), 3.97 (s, 2H), 3.86 (m, 1H), 3.71 (s, 2H), 2.91–2.73 (m, 2H), 2.06 (s, 2H). ^13^C-NMR (100 MHz, DMSO): δ 166.0, 154.5, 136.9, 136.8, 126.0, 118.2, 73.9, 67.7, 63.5, 49.0, 47.0, 44.2.

*Rivaroxaban* (**1**): 5-Chlorothiophene-2-carboxylic acid (1.3 g, 7.6 mmol) was treated with thionyl chloride (10 mL) at reflux for 2 h. After removal of the excess thionyl chloride by evaporation under vacuum, the residue was dissolved in anhydrous dichloromethane (20 mL). A mixture of compound **12** (1.1 g, 3.8 mmol) with TEA (5 mL) in anhydrous dichloromethane (40 mL) was added at 0 °C. After the completion of addition, the mixture was stirred for 30 min at the same temperature and another 2 h at room temperature. The mixture was filtered and the filter cake was washed with CH_2_Cl_2_ (15 mL × 3). The filter cake was dried under reduced pressure to give the rivaroxaban (**1**, 1.5 g, 92% yield) as a a white solid. ${\left[\text{α}\right]}_{\text{D}}^{21}$ −38.1°(*c* 4.3340, DMSO). ^1^H-NMR (400 MHz, DMSO): δ 8.98 (s, 1H), 7.69 (s, 1H), 7.56 (d, *J* = 8.3 Hz, 2H), 7.40 (d, *J* = 8.3 Hz, 2H), 7.19 (s, 1H), 4.84 (m, 1H), 4.19–4.17 (m, 3H), 3.97 (s, 2H), 3.83 (t, *J* = 7.3 Hz, 1H), 3.71 (s, 2H), 3.60 (s, 2H). ^13^C-NMR (101 MHz, DMSO): δ 166.0, 160.8, 154.1, 138.5, 137.1, 136.5, 133.3, 128.5, 128.2, 126.0, 118.3, 71.3, 67.7, 63.5, 49.0, 47.4, 42.2.

## 4. Conclusions

In conclusion, we have developed an efficient and facile synthetic method to synthesize rivaroxaban (**1**) in 39% global yield from (*R*)-2-(chloromethyl)oxirane over six steps using a Goldberg coupling reaction as a key step. To the best of our knowledge, this route is more straightforward and offers a lower cost of goods than previously reported syntheses of this drug.
